# Study protocol of psychometric properties of the Spanish translation of a competence test in evidence based practice: The Fresno test

**DOI:** 10.1186/1472-6963-9-37

**Published:** 2009-02-24

**Authors:** Josep M Argimon-Pallàs, Gemma Flores-Mateo, Josep Jiménez-Villa, Enriqueta Pujol-Ribera, Gonçal Foz, Magda Bundó-Vidiella, Sebastià Juncosa, Cruz M Fuentes-Bellido, Belén Pérez-Rodríguez, Francesc Margalef-Pallarès, Rosa Villafafila-Ferrero, Dolors Forès-Garcia, Josep Roman-Martínez, Esther Vilert-Garroga

**Affiliations:** 1 Divisió d'avaluació, Servei Català de la Salut, C/Travessera de les Corts, 131-159 Edifici Olímpia,08028 Barcelona, Spain; 2 Institut d'Investigació en Atenció Primària Jordi Gol (IDIAP Jordi Gol), C/Gran Via de les Corts Catalanes 587 àtic, 08007 Barcelona, Spain; 3 UD Barcelona Ciutat, Institut Català de la Salut, C/Sant Elies 42, 08006 Barcelona, Spain; 4 UD de Barcelonès Nord i Maresme, Institut Català de la Salut, C/La Plaça 93, 0830 Premia de Mar, Barcelona, Spain; 5 UD Centre, Institut Català de la Salut, C/Torrebonica s/n, 08227 Terrassa, Barcelona, Spain; 6 UD Tarragona, Institut Català de la Salut, C/Prat de la Riba 39, 43001 Tarragona, Spain; 7 UD Fundació Sant Pau i Santa Tecla, C/Joan Fuster s/n, 43007, Tarragona, Spain; 8 UD de FASS grup SAGESSA, Av President Companys s/n, 43201 Reus, Tarragona, Spain; 9 UD Costa de Ponent, Institut Català de la Salut, Autovia de Castelldefels, km 2, 7, 1a planta. 08907, L'Hospitalet de Llobregat, Barcelona, Spain; 10 UD de Palamòs, C/Hospital 36, 17230 Palamòs, Girona, Spain

## Abstract

**Background:**

There are few high-quality instruments for evaluating the effectiveness of Evidence-Based Practice (EBP) curricula with objective outcomes measures. The Fresno test is an instrument that evaluates most of EBP steps with a high reliability and validity in the English original version. The present study has the aims to translate the Fresno questionnaire into Spanish and its subsequent validation to ensure the equivalence of the Spanish version against the English original.

**Methods and design:**

The questionnaire will be translated with the back translation technique and tested in Primary Care Teaching Units in Catalonia (PCTU). Participants will be: (a) tutors of Family Medicine residents (expert group); (b) Family Medicine residents in their second year of the Family Medicine training program (novice group), and (c) Family Medicine physicians (intermediate group). The questionnaire will be administered before and after an educational intervention. The educational intervention will be an interactive four half-day sessions designed to develop the knowledge and skills required to EBP. Responsiveness statistics used in the analysis will be the effect size, the standardised response mean and Guyatt's method. For internal consistency reliability, two measures will be used: corrected item-total correlations and Cronbach's alpha. Inter-rater reliability will be tested using Kappa coefficient for qualitative items and intra-class correlation coefficient for quantitative items and the overall score. Construct validity, item difficulty, item discrimination and feasibility will be determined.

**Discussion:**

The validation of the Fresno questionnaire into different languages will enable the expansion of the questionnaire, as well as allowing comparison between countries and the evaluation of different teaching models.

## Background

Several systematic reviews have addressed the issue of the effectiveness of educational programs on Evidence Based Practice (EBP) in improving knowledge, skills and behaviour. In summary, these reviews, have consistently reported that EBP training results in improvements in participants' knowledge of methodological and statistical issues and enhances their attitudes towards the use of medical literature in clinical decision making, but no change in behaviour is found. [1-6] Nevertheless, these findings need to be interpreted with considerable caution as most of the studies had poor internal validity. [7] Furthermore, few authors provided detail on how the questionnaires were developed and validated, how the questionnaires were administrated and how long before the intervention.

Another systematic review assessed the available EBP teaching instrument methods.[8] They defined 3 levels of instruments, based on: (a) the type, extent, methods, and results of psychometric testing and (b) suitability for different evaluation purposes. Level 1 instruments are supported by established inter-rater reliability (if applicable), objective (non-self-reported) outcome measures, and multiple (≥ 3) types of established validity evidence (including evidence of discriminative validity). Therefore level 1 instrument are distinguished by the ability to discriminate between different levels of expertise or performance and are suited to document the competence of individual trainees. Furthermore, the robust psychometric properties in general support their use in formative or summative evaluations.

With the exception of the instruments classified as level 1[8], the rest of instruments should be used cautiously by educators to assess the EBP competence of individual trainees because they were developed to evaluate the effectiveness of specific curricula and lack evidence for discriminative validity. Within level 1 instruments the Fresno Test [9] and the Berlin Questionnaire [10] represent the instruments that evaluate most of EBP steps. The other instruments classified as level 1 evaluate a narrower range of EBP steps.

The Fresno questionnaire was designed to assess all domains of EBP.[9] It begins with the presentation of two scenarios that suggest clinical uncertainty. Short answer questions about the clinical scenarios require the candidate to formulate a focused question, identify the most appropriate research design for answering the question, show knowledge of electronic database searching, identify issues important for determining the relevance and validity of a given research article, and discuss the magnitude and importance of research findings. Unlike multiple choice or true-false questions, the open ended questions require examinees to show higher order thinking in response to an authentic task. These questions are scored by using a standardised grading system.

Table 1 shows the psychometric properties of both questionnaires. It can be concluded that both are reliable and valid for detecting the effect of instruction in EBP. More time and expertise are required to grade the Fresno Test. [8] The multiple-choice format of the Berlin Questionnaire restricts assessment to EBP applied knowledge but also makes it more feasible to implement. Nevertheless, there is another important difference between them: whereas the Fresno test was designed to assess all domains of EBP, not all skills in EBP are captured by the Berlin test (i.e., formulation of question, competencies in searching), which is concentrated more on the handling of research information. In taking the Fresno Test, trainees perform realistic EBP tasks, demonstrating applied knowledge and skills. For this reason we have chosen for translation the Fresno test.

**Table 1:** Properties of Berlin and Fresno test

**Test property**	**Measure used**	**Performance of Berlin test **[10]	**Performance of Fresno test **[9]
**Content validity **(test covers entire topic of interest)	Expert opinión	Expert opinion (five teachers in EBP)	Revisions based on experts' suggestions

**Inter-rater reliability **(degree to which 2 scorers rate a single performance similarly)	Inter-rater correlation	Total score 0.96 (IC 95%: 0.92–0.98)	Ranged from 0.76 to 0.98 for individual items, total scores 0.98

**Internal reliability **(degree to which all test questions on the test measure a single construct)	Cronbach's α average of all possible split half correlations	0.75	0.88

**Item difficulty **(relative difficulty of each item)	% of candidates who answer achieve a passing score	Not given	Ranged from moderate (73%) to difficult (24%); no easy items

**Item discrimination **(ability of each item to discriminate between those with overall high scores and those with overall low scores)	Item discrimination index (ranges from -1.0 to 1.0)	Not given	Ranged from 0.41 to 0.86, no items had negative or weak discriminations

**Construct validity **(evidence that the test measures the construct it intends to)	Mean scores of experts and novices compared by *t *test	Significant difference, higher expert scores than novices	Significant difference, higher expert scores than novices

*Adaptation of Ramos et al[9]

The primary aim of the study is the translation of the Fresno questionnaire into Spanish and its subsequent validation to ensure the equivalence of the Spanish version against the English original.

The specific objectives are: (a) To translate the test into Spanish using the back translation method; (b) To assess the construct validity of the test comparing the scores achieved by tutors with formal methodological training in EBP, the scores of a group of family medicine residents before they have had any formal training in EBP, and finally the scores of a group of family medicine residents with intermediate experience (training) in EBP; (c) To assess the responsive validity (change in knowledge and skills) of the test after a short course in EBP carried out in a group of family medicine residents; (d) To assess the reliability of the test by measuring the internal reliability (degree to which all test questions on the test measure a single construct), the inter-rater reliability (degree to which two scorers rate a single performance similarly), the intra-rater reliability (degree to which onescorer rate a single performance similarly); (e) To assess the feasibility of implementation by reporting the time required administering the test and reporting the time required scoring the instrument.

## Methods and design

The study comprises two stages: translation of the instrument into Spanish and its subsequent validation.

### Setting and context

The setting of the study will be the Primary Care Teaching Units in Catalonia (PCTU). At the beginning of the Family Medicine residence program (which longs four years) the medical residents are enrolled into a PCTU. Each PCTU comprises several health centers in a defined geographical area. There are seventeen PCTU in Catalonia.

The number of medical residents in each PCTU ranged from 2 to 52 in 2007. In the year 2007 there were 202 residents in their second year of training.

### Participants and study description

The instrument will be validated by administering it to a three groups: (a) the first group will comprise by tutors of family Medicine residents with formal methodological training in EBP and who participate regularly in randomised clinical trials conducted in primary care (expert group); (b) the second group will be formed by medical residents in their second year of the Family Medicine training programme in Catalonia, before they have had any formal training in EBP during their residence program (novice group); (c) finally, the questionnaire will be administered to a group of Family and Community Medicine physicians (intermediate experience).

In the present study the group of novices, as well as the group of intermediate experience will receive an educational intervention on EBP about four weeks after completing the pre-test. The last day of the course the test will be administered again allowing for the assessment of the responsiveness of the test. Since there are two groups with different experiences it could be expected that the responsiveness would be different, fostering the expansion of the result.

The following variables will be recorded for each participant: age, sex, year of graduation in medicine, courses in EBP completed prior to the educational intervention and time required in filling-in the test.

### The translation into Spanish

First of all, an e-mail letter will be sent to the original developers of the Fresno test asking permission to proceed with the translation and using of the tool for research purposes.

**Figure 1:**
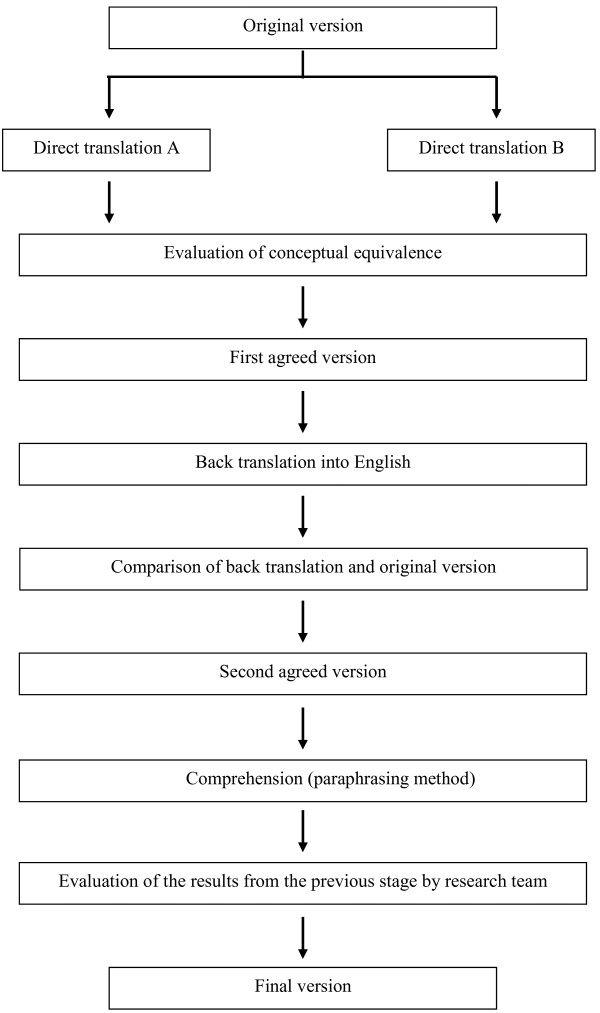
**Translation process of the Fresno questionnaire into Spanish**.

The test will be translated and back-translated according to guidelines for questionnaire adaptation in order to achieve the highest possible content validity [11,12] (Figure 1).

The original English version of the Fresno questionnaire will be translated into Spanish independently by two bilingual translators. A team made up of EBP experts, specialists in Family and Community Medicine and specialists in Preventive Medicine and Public Health, will review the translations. Based on the translations and the comments raised by the research team, an agreed version of the questionnaire will be obtained.

The next step will be to translate the agreed English version to make sure that it is conceptually equivalent to the original. Subsequently, the research team, with assistance from all the translators, we will compare the back translation with the original version in order to identify any questions that are not equivalent or which may be problematic.

Once the agreed version will obtained, a series of individual interviews will be conducted with residents and specialists in Family and Community Medicine to assess the understanding of the questionnaire (cognitive debriefing). The aspects that will be evaluated in the interviews are the degree of understanding of the items, the ease or difficulty in filling-in the questionnaire and the degree in which the format will be accepted. The interviews will be conducted with 5 residents and 5 specialists in Family and Community Medicine. The paraphrasing method will be used in the interviews, where the participants rephrase in their own words the items that present the greatest difficulty.

Finally, the research team will meet to evaluate the results of the questionnaire comprehension tests and obtain the final version of the pre-test.

### Educational intervention

The educational intervention that medical residents receive during their training program is an intensive and interactive four half-day session designed to develop the knowledge and skills required to practice evidence-based care. The audience is the residents performing the second year of the residence program. The course is compulsory for the residents in this specialty. Regarding the present study, lecturers will be advised not to modify their sessions with a view to coaching for the test.

The EBP course includes a series of short lectures and small group facilitate workshops design to teach the principles and practice of evidence based medicine. The emphasis is on practical and efficient point-of-care information retrieval, appraisal, and application at the bedside. At the end of the instruction an evaluation is mandatory. Until now the assessment had been a multiple choice questionnaire.

The course is modelled after the steps of EBP first described in 1992 by Cook DJ, et al.[13] Sessions featured a mix of, interactive lectures, workshops and case-based studies around six topics: Writing a clinical question; searching the medical literature; Selecting and obtaining the evidence; Critical appraisal of systematic reviews, randomised clinical trials (RCTs) and diagnostic test; Interpreting the clinical relevance and precision of the results; Application of evidence to clinical care.

### Administration of the test

To allow the assessment of responsiveness the test will be administered before and after the educational intervention. Repeated administration of measures, particularly those focussing on knowledge, may over-estimate the educational effect and therefore the responsiveness of the test. To minimise the recall bias two sets of test with different scenarios will be prepared. Moreover the pre-test will be administered four weeks before starting the educational module.

Novice and intermediate experience participants will be invited four weeks before starting the educational module to attend a conference about research in primary care and the importance of applying the results of this research into clinical practice. The project of validation the Fresno test will be also presented. The sessions will be decentralised by Health Regions. At the end of each session participants will receive the test (before test). The other test will be administered again on the last day of the course (after test).

The members of the expert group belong to a network of physicians who are regularly engaged in the design and conduct of clinical trials. The network holds in November its annual conference. The members who attended the conference will be asked for filing-in the test. In order to assess the equivalence of the two sets of the questionnaire, both sets will be randomly assigned among the folders distributed to the participants.

In order to assess the feasibility of the test the time required for filling-in the test will be recorded in all cases.

All the participants will be explicitly informed about the research character of the test and that participation was voluntary.

### Scoring the test

To score the test the forms will be encoded and the personal identification data removed from all the participants. Two scorers will score independently the test. They will be blind to the identity of the participants, but not if the questionnaire is given before or after the educational intervention. Previously they will perform a pilot test scoring with ten tests to agree with the scoring methodology.

### Construct validity

To assess the construct validity of the test the expertise of the participants will be used as an important indicator for knowledge and skills in EBP. It was hypothesized that those with more experience based on theoretical grounds would have higher scores in the test. The scores achieved by the experts will be compared with the scores obtained by the novice group and the intermediate experience group (before test), using an analysis of variance. If a significant difference will be found, a post hoc analysis by Scheffé's method will be added.

Furthermore, residents will be asked to inform their real exposure to EBP measured as number of courses, seminars and conferences have attended and how many hours each course, seminar and conference have comprised. The relationship between the hours of exposure and the score of the test will be assessed by using Spearman's rank correlation coefficient.

### Responsiveness

Responsiveness is the extent to which instruments are sensitive enough to detect the smallest difference considered clinically relevant. [14] Responsiveness is a special type of construct validity.

To calculate the responsiveness of a test and to inform it to the readers is of great importance when planning a clinical trial. Indeed, the more responsive an instrument, the smaller is the number of the patients required to achieve statistical power, or alternatively, the more power is achieved with the same sample size.

Responsiveness statistics used in the analysis will the effect size, the standardised response mean, and Guyatt's method.

The effect size is the difference between the mean baseline and follow-up scores on the measure, divided by the standard deviation of the baseline scores. The effect size is defined as "small" (E-S < 0.2), "small to moderate" (E-S between 0.2 and 0.5), "moderate to large" (E-S between 0.51 and 0.79), "large" (E-S > 0.79), as suggested by Cohen.[15] The results were categorized by group of experience (novice and intermediate).

The Standardised Response Mean (SRM) is calculated as the mean change in scores divided by the standard deviation of these changes. Again the results were categorized by group of experience.[16]

The formula for Guyatt's responsiveness index is defined as Δx/√2 × MSEx, where Δx is the minimally clinically important change on the measure and MSEx is the mean squared error of the variable obtained from an analysis of variance model that examines repeated observations of the measure in clinically stable subjects.[17] Alternatively, if there are only two observations of the measure, MSEx is the standard deviation of the individual change scores in clinically stable patients (i.e., placebo-treated patients). In the present study we assume that the most stable participants were those in the expert group. This index is sometimes referred to as the modified standardised response mean, or to as the index of responsiveness.[16]

### Internal consistency

Internal consistency refers to the extent to which individual items of the instrument are consistent to one another and reflect an underlying scheme or construct. For internal consistency reliability, two measures will used: Cronbach's alpha and corrected item-total correlations.

Cronbach's alpha coefficient is used to evaluate the internal consistency between the questionnaire elements. Cronbach's alpha coefficient measures how well a set of items (or variables) measures a single uni-dimensional latent construct. Achievable values for Cronbach's alpha range from 0 (signifying no correlation) to 1 (indicating identical results). When data have a multidimensional structure, Cronbach's alpha will usually be low. Technically speaking, Cronbach's alpha is not a statistical test – it is a coefficient of reliability (or consistency).[18]

The reliability of an instrument has implications for whether it is suitable for application in group or individual evaluation. For the evaluation of individuals high levels of reliability, above 0.90, have been recommended. For group comparisons, levels over 0.70 are recommended. In the present study, values greater than 0.70 will be considered evidence of internal consistency.[19]

Pearson's correlation coefficient will be also calculated to assess the relation between an individual item and the instrument as a whole omitting that item (corrected item total correlation) to identify items contributing to a low reliability. Corrected item-total correlations indicate the extent to which each item relates to the construct measured by the total score. Correcting the total score by removing the item of interest prevents spuriously high values due to item overlap; values greater than 0.30 will be considered evidence of internal consistency. [20]

### Item difficulty

We will assess the difficulty of each question by calculating the proportion of correct answers for each question. Item difficulty is important because it reveals whether an item is too easy or too hard. In either case, the item may add to the unreliability of the test because it does not aid in differentiating between participants. Wide range of difficulties allows a test to be used with both expert and novice groups.

The optimal item difficulty depends on the question-type. Nevertheless, scores per question by course participants should not fall below 0.1 or go above 0.9, as scores outside these parameters do not tend to provide additional information to distinguish the more knowledgeable participants from the less knowledgeable ones. Such items should either be revised or replaced.

We will analyse item difficulty only in the questions with two alternatives: true and false.

### Item discrimination

The single best measure of the effectiveness of an item is its ability to separate participants who vary in their degree of knowledge of the material tested, and their ability to use it. If one group of students has mastered the material and the other group had not, a larger portion of the former group should be expected to correctly answer a test item. Item discrimination is the difference in proportions for test takers answering correctly between those scoring in the upper 27% on total score and those scoring in the lower 27%. The following levels were used as a guideline for acceptable items: 0–24% unacceptable; 25%–39% good item; 40–100% excellent item.

### Inter-rater reliability

The inter-rater reliability is defined as the degree to which two scorers rate a single a single performance similarly. Inter-rater reliability will be tested using Kappa coefficient for qualitative items and intra-class correlation coefficient for quantitative items and the overall score.

### Equivalence of sets 1 and 2

Equivalence of sets 1 and 2 of the test will be determined by calculating the intra-class correlation coefficient of the overall score for the experts.

### Feasibility

The time required for filling-in the test and the time required for scoring the test will be described using the mean, standard deviation, median and range inter-quartile.

### Floor and ceiling effects

The presence of floor and ceiling effects may influence the reliability, validity and responsiveness of an instrument. An intervention effect might be missed for people who occupy the maximum score. In order to determine floor and ceiling effects, we will calculate the percentage of patients with very low and very high scores. Since there is no consensus on how to define floor and ceiling effects mathematically,[21] we have determined *a priori *that floor and ceiling effects were considered present when scores were found lower than 10, and higher than 200, respectively.

### Exclusions of the analysis

Several cases will be excluded of the analysis: the resident who do not fill-in the pre-test; any residents who fail to answer the post-test were not taken into account for the responsiveness analysis, as well as those residents who do not attend at least three half-days of the EBP course.

### Statistical packages

Statistical analyses will be conducted by using Stata software version 9.0 (STATA Corp, College Station, TX) and with SPSS software version 15.0 for Windows (SPSS, Chicago, IL, USA). Effect size calculations will be performed by using the effect size generator software program version 3.2 (Effect size generator).

## Discussion

Reliability, validity and responsiveness are context-specific attributes, and an instrument that has demonstrated satisfactory measurement properties in one population is not necessarily appropriate for use in other populations.[22] The validation of the Fresno questionnaire into different languages, professional groups, and cultural settings, will enable the generalisability of the test, as well as allowing comparisons between countries and the evaluation of different teaching methods.

Ramos et al. [9] pointed out that there are limitations to the validity, reliability, and general utility of the Fresno test. The reason being the groups they used to develop and validate the test, which probably represented the extremes of proficiency, leaving the middle ground relatively under-represented. The properties of the test may change when it used to assess groups of people that are more representative of the full range of proficiency in EBP. In the present study the inclusion of the group with intermediate experience may represent a wider range of proficiency in EBP. the validation of the Fresno questionnaire into Spanish, in a population with a different background of experience in EBP than the population recruited by Ramos et al [10], will enable the generalisability of the test, as well as allowing comparisons between countries and the evaluation of different teaching methods.[9]

The goals of the instrument include differentiating between people who are knowledgeable and skilled in EBP from those who do not at a point in time (a discriminative instrument) as well as measuring how much knowledge and skills have changed during a period of time (an evaluative instrument). In both cases the instrument must have a high ratio of signal to noise (reliability and responsiveness, respectively) and be valid.

Ramos et al. [9] did not perform an assessment of the responsiveness of the test. In the present study the group of novices, as well as the group of intermediate experience received an educational intervention on EBP about four weeks after completing the pre-test. The last day of the course the test was administered again allowing for the assessment of the responsiveness of the test. Since there are two groups with different experiences it could be expected that the responsiveness would be different, fostering the generalisability of the result.

A lack of clarity exists about the definition and adequate approach for evaluating responsiveness. Terwee et al. [16] presented an overview of different categories of definitions and methods used for calculating responsiveness identified through a literature search. Twenty-five definitions and 31 measures were found. In this study, three commonly used responsiveness indices will be estimated. Mean change scores in the Fresno test were calculated by subtracting the baseline score from the data obtained after the educational intervention: the effect size (ES) the Guyatt's responsiveness statistic (GRS), and the standardized response mean (SRM). The responsiveness of a particular measure may be influenced by the responsiveness index used, irrespective of the scope of the measure or the direction of the change. Whereas most indices use the mean change of the score over time, there are significant differences in how the standard deviations or variability in the data is used in the calculation. For example, the GRS is calculated using the standard deviation of the change scores among subjects who had stable scores (in our study the expert group), whereas the SRM uses the standard deviation of the change scores. It is therefore possible that significant differences could exist in the variability in the selected subgroups, resulting in differences in the perceived responsiveness of the measure depending upon the responsiveness index chosen.

Strength of this study is the assessment of the feasibility of the test. Feasibility concerns the ease of administration and processing of an instrument. These are important considerations for staff and researchers who collect and process the information produced by instruments.[23] Instruments that are difficult to administer and process may jeopardise the conduct of research and disrupt educational efforts. An obvious example is the additional resources required for interviewer administration over self-administration. The complexity and length of an instrument will have implications for the form of administration. Staff training needs must be considered before undertaking administration. Finally, staff attitudes and acceptance of instruments can make a substantial difference to respondent acceptability.

The results and interpretation of this study should be considered in light of several potential limitations. Most notably, we used a before-after design. This is a shortcoming either for assessing the responsiveness of the test since we did not have the variability of a control group and we had to use as a surrogate the standard deviation of the expert group, and also for assessing the effectiveness of the course. Furthermore, the intensity of the intervention and the period chosen to minimise the recall accuracy of the participant (four weeks) may also influenced the responsiveness indices, due to the effect of external influences such as attendance to another course, looking into the main topics of the test in a book or simply by exchanging opinions and knowledge with other residents or their tutors.

Evaluation of educational interventions should not only be concerned about a gain in knowledge and skills, but also on how this gain is transferred to workplace (behavioural change) and impact on patients (health outcomes). Regarding the effectiveness of the educational intervention, assessment of EBP skills acquisition will be performed immediately after the course; so long term outcomes will not be measured. Moreover, the Fresno Test was not developed for measuring behavioural change, and the present study has not been designed either for assessing even short term behavioural change. Therefore we will not be able either to draw conclusions on behavioural change after the educational intervention.

For study-logistic reasons, only residents and tutors of Family Medicine will be included in the validation study. While it could be suggested that more similarities than differences exist between the different groups of medical residents with regard to EBP issues, the use of Fresno test in a population including residents from other specialties would require testing for validity and reliability in the specific resident's group. Levels of inter-rater reliability, internal consistency and discrimination are intimately dependent on the population which has taken the test) and it cannot be assumed that any of these key attributes can be maintained in subsequent studies with a population with a different background experience in EBP.

The review of Shaneyfelt et al.[8] identified 104 unique instruments for evaluating education in evidence-based practice. Such a large number can only serve to confuse educators and researchers choosing an instrument for applications including clinical trials and teaching. In front of this proliferation of instruments, many of which do not adequately draw on recommended criteria for instrument development, it could be recommend that no further instruments be developed, and that the efforts of researchers should be directed towards refining and validating existing instruments. The validation of the Fresno questionnaire into different languages, professional groups, and cultural settings, will enable the expansion of the test, as well as allowing comparisons between countries and the evaluation of different teaching methods

## Competing interests

The authors declare that they have no competing interests.

## Authors' contributions

JAP and GFM are the principal investigators responsible for the conception of the project and drafting the manuscript. JJV and GFM will be in charge of the statistical analyses. GF, MBV, SJ, MCFB, BP, FMP, RVF, DFG, JRM contributed to the description of the background and general design. All authors have read and approved the final manuscript.

## Pre-publication history

The pre-publication history for this paper can be accessed here:



## References

[B1] Norman GR, Shannon SI (1998). Effectiveness of instruction in critical appraisal (evidence-based medicine) skills: a critical appraisal. CMAJ.

[B2] Green ML (1999). Graduate medical education training in clinical epidemiology, critical appraisal, and evidence-based medicine: a critical review of curricula. Acad Med.

[B3] Taylor S, Muncer S (2000). Redressing the power and effect of significance. A new approach to an old problem: teaching statistics to nursing students. Nurse Educ Today.

[B4] Parkes J, Hyde C, Deeks J, Milne R (2001). Teaching critical appraisal skills in health care settings. Cochrane Database Syst Rev.

[B5] Coomarasamy A, Khan KS (2004). What is the evidence that postgraduate teaching in evidence based medicine changes anything? A systematic review. BMJ.

[B6] Flores-Mateo G, Argimon JM (2007). Evidence Based Practice Education in Postgraduate Health Care: A Systematic Review. BMC Health Serv Res.

[B7] Hatala R, Guyatt G (2002). Evaluating the teaching of evidence-based medicine. JAMA.

[B8] Shaneyfelt T, Baum KD, Bell D, Feldstein D, Houston TK, Kaatz S (2006). Instruments for evaluating education in evidence-based practice: a systematic review. JAMA.

[B9] Ramos KD, Schafer S, Tracz SM (2003). Validation of the Fresno test of competence in evidence based medicine. BMJ.

[B10] Fritsche L, Greenhalgh T, Falck-Ytter Y, Neumayer HH, Kunz R (2002). Do short courses in evidence based medicine improve knowledge and skills? Validation of Berlin questionnaire and before and after study of courses in evidence based medicine. BMJ.

[B11] Beaton DE, Bombardier C, Guillemin F, Ferraz MB (2000). Guidelines for the process of cross-cultural adaptation of self-report measures. Spine.

[B12] Guillemin F, Bombardier C, Beaton D (1993). Cross-cultural adaptation of health-related quality of life measures: literature review and proposed guidelines. J Clin Epidemiol.

[B13] Cook DJ, Jaeschke R, Guyatt GH (1992). Critical appraisal of therapeutic interventions in the intensive care unit: human monoclonal antibody treatment in sepsis. Journal Club of the Hamilton Regional Critical Care Group. J Intensive Care Med.

[B14] Liang MH, Lew RA, Stucki G, Fortin PR, Daltroy L (2002). Measuring clinically important changes with patient-oriented questionnaires. Med Care.

[B15] Cohen J (1977). Statistical power analysis for the behavioral sciences.

[B16] Terwee CB, Dekker FW, Wiersinga WM, Prummel MF, Bossuyt PM (2003). On assessing responsiveness of health-related quality of life instruments: guidelines for instrument evaluation. Qual Life Res.

[B17] Guyatt G, Walter S, Norman G (1987). Measuring change over time: assessing the usefulness of evaluative instruments. J Chronic Dis.

[B18] Bland JM, Altman DG (1997). Cronbach's alpha. BMJ.

[B19] Streiner D, Norman GR (2003). Health measurement scales A pracitcal guide to their development and use.

[B20] Norman GR, Sloan JA, Wyrwich KW (2003). Interpretation of changes in health-related quality of life: the remarkable universality of half a standard deviation. Med Care.

[B21] Terwee CB, Bot SD, de Boer MR, Windt DA van der, Knol DL, Dekker J (2007). Quality criteria were proposed for measurement properties of health status questionnaires. J Clin Epidemiol.

[B22] Fitzpatrick R, Davey C, Buxton MJ, Jones DR (1998). Evaluating patient-based outcome measures for use in clinical trials. Health Technol Assess.

[B23] Erickson P, Taeuber RC, Scott J (1995). Operational aspects of Quality-of-Life Assessment. Choosing the right instrument. Pharmacoeconomics.

